# The Role of DACT Family Members in Tumorigenesis and Tumor Progression

**DOI:** 10.7150/ijbs.70784

**Published:** 2022-07-11

**Authors:** Yu Zeng, Jiqin Zhang, Jianhe Yue, Guoqiang Han, Weijia Liu, Lin Liu, Xin Lin, Yan Zha, Jian Liu, Ying Tan

**Affiliations:** 1 Department of Neurosurgery, Guizhou Provincial People's Hospital, Guiyang, China.; 2 Department of Anesthesiology, Guizhou Provincial People's Hospital, Guiyang, China.; 3 Department of Nephrology, Guizhou Provincial People's Hospital, Guiyang, China.; 4 Department of Neurosurgery, The Second Affiliated Hospital of Chongqing Medical University, Chongqing, China.; 5 Department of Respiratory and Critical Care Medicine, Guizhou Provincial People's Hospital, Guiyang, China.

**Keywords:** DACT family members, Cancer, Proliferation, Apoptosis, Invasion, Prognosis

## Abstract

Disheveled-associated antagonist of β-catenin (DACT), which ubiquitously expressed in human tissue, is critical for regulating cell proliferation and several developmental processes in different cellular contexts. In addition, DACT is essential for some other cellular processes, such as cell apoptosis, migration and differentiation. Given the importance of DACT in these cellular processes, many scientists are gradually interested in studying the role of DACT in tumorigenesis and cancer progression. This review article focuses on the latest research regarding the essential functions and potential DACT mechanisms in the occurrence and progression of tumors. Our study indicates that DACT may act as a tumor biomarker for cancer diagnosis and prognosis, as well as a promising therapeutic target in cancers.

## Introduction

It is well known that cancer remains the leading cause of human death, which results from accumulated genetic and epigenetic alterations of various cancer-related genes, including tumor suppressor genes (TSGs) and oncogenes 1-3. TSGs are considered as crucial genes that are sufficient to control the growth of tumors and regulate many cellular biological processes, including induction of apoptosis and autophagy, inhibition of cell proliferation, suppression of invasion, and DNA damage repair 4-6. Indeed, the inactivation of TSGs is an important mechanism contributing to tumorigenesis and the progression of cancer 7, 8. Although more and more TSGs have been found and various therapies targeting these TSGs also have been developed over the past several decades, the prognosis of cancers remains poor. Therefore, it is urgent to identify new therapeutic targets and potential molecular mechanisms that regulate the development of cancer.

DACT family members (DACTs) were initially isolated from yeast-two hybrid screens with the Dishevelled (Dvl) PDZ domain as bait 9-11. In humans, it has been reported that DACTs comprise three functional members: DACT1, DACT2, and DACT3, which share some conserved domains 12. The DACTs encode a vital group of adaptor proteins that physically interact with different factors to maintain development and postnatal homeostasis 13. As adaptors, DACTs exert cell biological function by regulating key signaling pathways, such as Wnt, TGF-β, YAP, and NF-κB signaling pathways (as summarized in Table 1) 14-19. In recent years, accumulating evidence has revealed that DACTs are aberrantly expressed in many malignant tumors and associated with unfavorable survival. This review will discuss the possible function and underlying mechanisms of DACTs in tumor development and their values in cancer diagnosis and prognosis.

## Structural and biological functions of DACT family members

DACTs are intracellular proteins that can bind to many molecules in the cytoplasm and nucleus 20. All members are characterized by a PDZ-binding motif at the C terminus, a leucine zipper motif near the N terminus (leucine zipper, LZ), and two serine-rich domains (one in the C-terminal region and the other right after the leucine-zipper domain) 21, 22. Studies have shown that structures and functional levels of DACTs are highly conserved 20. Based on research at the amino acid sequence level, there are 799-aminoacids for DACT1 protein, 774-aminoacids for DACT2 protein, and 610-aminoacids for DACT3 protein 23, 24. DACT1 and DACT2 share 28.8% total amino-acid identity. DACT3 is approximately 27% similar to DACT1 and 24% similar to DACT2 12. However, DACT1 are more similar to DACT3 than to DACT2 at the C-terminus 21. At the cellular level, DACT1 is found throughout the cytoplasm as punctate spots and diffusely in the nucleus, while DACT2 protein locates mainly in the cytoplasm, and DACT3 is predominantly in the nucleus 14, 15, 25. It has been previously shown that DACT1 and DACT2 are the nucleocytoplasmic shuttling proteins, containing both nuclear localization signal (NLS) and putative nuclear export signal (NES) 15, 26. Immunofluorescent staining revealed that the N-terminal region of is totally localized in the cytoplasm and the C-terminal region settled in the nucleus, which suggested that NES and NLS are contained in N-terminal and C-terminal region, of the DACT1 or DACT2, respectively 15, 26. It is worth noticing that the bioinformatic analysis found that there is a typical NES in N-terminal regions and at least one NLS in C-terminal regions of DACT3, meaning DACT3 may also be nucleocytoplasmic shuttling proteins like DACT1 and DACT2^15^. Thus, similarities and differences among the three DACTs suggest that each DACT paralog not only maintains conserved roles, but also has divergent functions in signal transduction.

DACTs play a critical role in the regulation of cellular function and vertebrate development. As reported previously, DACTs expression has been associated with the development of most organs and tissues, including the teeth, eye, heart, brain, and kidney 9, 27-30. Additionally, DACT1 was reported to mediate TGF-β1-induced apoptosis of mesangial cells 31. It has been also demonstrated that DACT1 downregulation facilitates proliferation and neuronal differentiation of neural stem cells 32. Moreover, DACT1 was proved to be a novel atrial fibrillation-related gene by regulating connexin43 via cytoskeletal organization induced by β-catenin accumulation in cardiomyocytes 33. Another study showed that the deletion of DACT2 significantly increases the proliferation rates of mouse embryonic fibroblasts cells during tooth development 20. Besides, DACT3 has been reported to inhibit Wnt-induced epithelial-to-mesenchymal transition (EMT) in renal tubular cells 34. Considering the fact that DACT2 is involved in so many cellular activities, it is natural to imagine that changes of their expression may lead to the occurrence of pathological conditions Indeed, Human DACT1, 2, and 3 genes are located on chromosomes 14q22.3, 6q27, and 19q13.32 region, respectively. Notably, the abnormalities in these regions are often tightly related to the occurrence of tumors 12, 14, 23, 24, 35.

## DACTs in cancer development

### DACT1 and cancer

Among three members in the DACT family, DACT1 is the best investigated in various types of cancer. Wang et al. reported that DACT1 was down-expressed in primary gastric cancers 19. Meanwhile, low expression of DACT1 was proved in primary gastric cancer tissues compared with the adjacent nontumor tissues 19. In bladder urothelial carcinoma, DACT1 protein was decreased or absent in bladder cancer tissues 36. Conversely, high DACT1 expression was observed in almost all normal bladder samples 36. A similar result was found in breast cancer, that DACT1 expression was significantly silenced or downregulated in cancerous tissues compared with paired surgical-margin tissues and normal breast tissues 25. Further analysis based on the gene expression-based outcome for breast cancer online database showed that decreased expression of DACT1 was associated with estrogen receptor negative and higher histologic grade 25, indicating that DACT1 could be considered as a potential indicator for different histological subtypes of breast cancer. DACT1 was also significantly downregulated in cervical cancer tissues compared with its expression in normal tissues 37, 38. Consistent with previous studies, DACT1 expression showed an obviously significant difference between either normal ovarian tissues or benign lesions and malignant tissues 39. However, DACT1 was reported to be overexpressed in colon cancer and squamous cell carcinoma 40, 41, which indicates that the biological function of DACT1 varies among malignancies.

In addition, researchers reported that the expression of DACT1 was significantly down-regulated in non-small cell lung cancer tissues (NSCLC) compared with that in normal lung tissues 42. Patients with low expression of DACT1 were significantly associated with poor prognosis 42. Moreover, decreased expression was positively correlated with poor differentiation, high pathologic TNM stage, and lymph node metastasis of lung cancer 42. However, in esophageal squamous cell carcinoma (ESCC), it demonstrated that DACT1 expression was increased compared with that in normal squamous cell epithelia 41. Higher DACT1 expression was also shown to be significantly correlated with regional lymph node metastasis and P-stage 41. All of these evidences strongly indicate that DACT1 may serve as a predictive factor for the prognosis of cancer, and the function of DACT1 may be dependent on cellular context.

### DACT2 and cancer

Previous studies have reported that DACT2 expression was significantly depressed in primary breast cancer samples compared with adjacent normal breast tissues 35, 43. Significant down-regulation of DACT2 expression was also observed in NSCLC tissues, colorectal cancer and hepatocellular carcinoma (HCC) tissues compared to normal tissues 44-48. Additionally, the expression level of DACT2 was significantly reduced in primary esophageal cancer samples compared with adjacent normal esophageal mucosa, and further statistical analysis showed that the frequency of DACT2 silencing significantly correlated with differentiation and prognosis of ESCC 49-51. Moreover, the decreased expression of DACT2 is closely correlated with the invasion, metastasis, occurrence, and development of gastric cancer, prostate, and papillary thyroid cancer 52-55. Similarly, based on our findings, DACT2 was significantly down-expressed in glioma tissues compared with normal brain tissues and the paired adjacent tissues 16. Expression levels of DACT2 in glioma tissues significantly correlated with the WHO grade, Karnofsky Performance Score and age^16^. Lower DACT2 expression had a significantly poorer overall survival compared to patients with higher DACT2 expression in gliomas with different grades 16.

### DACT3 and cancer

Compared with DACT1 and 2, DACT3 has been less well characterized, and its function in tumorigenic signaling and development remains unclear. Recent research revealed that DACT3 expression was reduced in NSCLC tissue, which was correlated with lymph node metastasis and poor prognosis of NSCLC 56. It also demonstrated that the expression of DACT3 is also consistently reduced in colorectal cancer 14. Further analysis showed DACT3 expression was related to prognosis and validated to be associated with the pathological stage of colon cancer 57. Interestingly, Luo et al. performed the weighted gene co-expression network analysis to identify the key modules and hub genes in bladder cancer, and they found that three hub genes (DACT3, TNS1, and MSRB3) were related to lymph node metastasis and prognostic of bladder cancer, which might provide new insights into the therapeutic target of bladder cancer 58.

Together, all these studies demonstrated that the association between DACTs expression and outcome in most types of cancers strengthened the importance of the role of DACTs in tumor progression and validated their potential value as a robust prognostic biomarker. The expressions of DACTs in cancers are shown in Table 1.

## Expression regulation of DACT family members in cancers

### Transcriptional and Posttranscriptional regulation of DACTs

Both transcriptional and posttranscriptional regulation play crucial roles in DACTs expression in cancers. MicroRNAs (miRNAs) are small non-coding RNAs that have been identified to play pivotal roles in various biological contexts by mediating the regulation of target gene expression at the posttranscriptional level 59-67. In HCC, Gan et al. revealed that DACT1 might act as a target gene of miR-1269 by bioinformatics analysis 68. Besides, miR-324-3p was demonstrated that it cloud directly target DACT1 and negatively regulate its expression in HCC cells 66. Furthermore, rescue experiments revealed that DACT1 could reverse the effects of miR-324-3p in HCC cells 66. Recently, evidence showed that miR-324-3p targeted the DACT1 transcript directly at its 3′UTR region 37. However, lncRNA H1FX-AS1 modulates miR-324-3p-mediated inhibition of DACT1 in cervical cancer 37. MiRNAs are also involved in the regulation of DACT2 expression. Zhao et al. showed that expression of miR-214 is elevated, but the expression of DACT2 mRNA is decreased in gastric cancer tissues, being closely correlated with the invasion, metastasis, occurrence, and development of gastric cancer 52. Furthermore, miR‑214 can directly bind with the 3'‑UTR seeding region of DACT2 mRNA to regulate its expression 52. Similarly, it was indicated that the expression of DACT2 increased in breast cancer tissues accompanied by decreased expression of miR-503-3p 69. Meanwhile, DACT2 was further proved to be a direct target of miR-503-3p 69. Recently, Wang et al. reported that miR-181a inhibited DACT2 by downregulating mixed-lineage leukemia 3(MLL3) expression in human umbilical vein endothelial cells, resulting in papillary thyroid cancer progression 70. As a member of DACTs, the expression of DACT3 is also modulated by miRNAs in cancers. Ren et al. found a significantly negative correlation between DACT3 expression and miR-638 expression in ESCC and breast cancer tissues 71. Importantly, it was further demonstrated that miR-638 might negatively regulate the expression of DACT3 by targeting the 3′UTR region of DACT3 to promote malignancy 71. Interestingly, cigarette smoke cloud induces expression of miR-31, which directly interacts with DACT-3 to decrease the expression of DACT3 in lung cancer cells 72 (as summarized in Table 2).

Transcriptional level regulation is another primary method involved in DACTs expression regulation. Esposito et al. demonstrated that DACT1 was transcriptionally regulated by TGF-β to modulate Wnt signaling 73. A recent study demonstrated that the rs9364433 single nucleotide polymorphism in gene promoter has an allele-specific effect on DACT2 expression modulated by transcription factor TFAP2A 44. The G allele is associated with diminished TFAP2A binding leading to the transcriptional suppression of the DACT2 gene in NSCLC cell lines and tissues 44.

### Epigenetic regulation of DACTs

Genetic and epigenetic variation work in concert to influence human health and disease 74. Distinct from genetic mutation, epigenetic influences modify gene expression without permanent changes in the genomic sequence 74-76. Indeed, recent studies demonstrated that epigenetic alterations are considered the main regulation mechanisms during carcinogenesis and cancer progression. The epigenetic modifications can be generally categorized into DNA methylation, histone proteins modifications, and mutations in chromatin remodeling complexes 76. Notably, DNA methylation is the most extensively studied epigenetic mechanism that predominantly occurs in CpG islands (CGIs), preferentially located at the 5′ promoter region of more than 50% of human genes 75, 77, 78. Hypermethylation of TSGs in CGI of the promoter regions is an alternative mechanism for TSG silencing and could occur early in tumorigenesis, thus serving as a promising tumor marker for diagnosis of multiple cancers 25.

Silencing of DACT family members can also be occurred by methylation of CpG islands in the promoter region, which plays an important role in tumorigenesis and development of tumors. For example, Yang et al. reported that the DACT1 gene was inactivated by hyper-methylation in the promoter region in nasopharyngeal carcinoma with 5-aza-deoxycytidine treatment, which can be reactivated by demethylation 79. Promoter CpG methylation of DACT1 was also detected in breast cancer tissues, which was correlated with its downregulation 25. Demethylation treatment of breast cancer cell lines restored expression of DACT1 along with promoter demethylation, as well as in hepatoma cell lines 80, suggesting that promoter methylation is a major mechanism for DACT1 silencing in breast cancer cells 25. In addition, methylation of the DACT1 CpG island is common in bladder cancer, repressing DACT1 expression 36. DACT1 gene hypermethylation was closely related to tumor size, grade, and stage, which indicates hypermethylation of DACT1 may be a potential prognostic factor progression of bladder urothelial carcinoma 36. Based on a large-scale gene sequencing analysis of gastric cancer patients, it was found that different methylated levels of DACT1 promoter were identified in the gastric cancer tissues while unmethylated in normal gastric mucosal tissues 81. Notably, three methylated CpG sites (CpG-515, CpG-435, and CpG-430) of DACT1 promoter were significantly associated with the poor survival of gastric cancer patients 81. Similarly, Deng et al. demonstrated that gene silence of DACT1 was mediated by promoter methylation in gastric cancer cells, and DACT1 methylation was significantly associated with tumor metastasis, invasion, and advanced tumor stage 19.

According to previous reports, DNA methylation played a crucial role in the silencing of DACT2. Guo et al. found that DACT2 was frequently methylated in human ESCC, which may be one of the main mechanisms for DACT2 inactivation 50. It also demonstrated that DACT2 was silenced by promoter hypermethylation in HCC, contributing to cancer progression 46. Additionally, DACT2 was reported to be frequently methylated in human lung cancer, and methylation of DACT2 was associated with poor differentiation of lung cancer 45. The similar results were reported in papillary thyroid cancer 54, breast cancer 35, 82, 83, esophageal cancer 50, 51, gastric cancer 53, colorectal cancer 84 and nasopharyngeal cancer 85. However, it was shown that promoter methylation of DACT1 and DACT2 may not be a common event in oral squamous cell carcinoma 86, suggesting promoter methylation of DACTs has the tumor cell-specific, and the precise mechanism of this gene family inactivation need to be further studied.

DACT3 expression is also frequently reduced in cancers. However, promoter methylation may not be the primary mechanism that mediates DACT3 silencing. According to the research, histone modification may be the main regulated mechanism for the inactivation of DACT3 in colon cancer 14. These results reinforce the importance of epigenetic regulation as a major mechanism of DACTs silencing in tumor progression. As shown in Table 3, the methylation status of DACTs is summarized.

## DACT family members regulate cancer cell proliferation and cell cycle

Uncontrolled proliferation is characteristic of cancer cells and represents one of the hallmarks of neoplastic growth 87. The cell proliferation inhibition capacity of DACTs is discovered in various cancers including gastric cancer 19, 52, 53, 88, leukemia 89, breast cancer 25, 35, 69, 82, 83, cervical cancer, HCC 46, 66, lung cancer 45, 56, papillary thyroid cancer 54, 70, esophageal cancer 50, 51, 71, 90, glioma 16, nasopharyngeal carcinoma 85, 91. Mechanically, DACTs exert their proliferation‐inhibitory effects through changes in signaling pathways. Zhu et al. demonstrated that DACT1 overexpression led to a strong cell cycle arrest at the G0/G1 phase through inhibiting Wnt/β-catenin signaling by reducing nuclear β-catenin levels, which resulted in KG-1α cell proliferation inhibition 89. Yin et al. found that DACT1 reduced the expression of active β-catenin and its downstream target gene c-MYC in breast cancer cells, thus inhibiting breast cancer cell proliferation 25. In addition, Wang et al. reported that DACT1 suppressed gastric cancer cell proliferation via decreasing the expression of NF-κB signaling downstream factors, including oncogenic interleukin-8 (IL-8) and tumor necrosis factor-α (TNF-α) 19. DACT1 was also proved that interact with proteins to regulate cell proliferation. Gao et al. found that DACT1 was a cyclin G2-interacting protein that was required for the cyclin G2-mediated inhibition of β-catenin expression 88. Cyclin G2 inhibited the activity of CKI to phosphorylate DACT1, causing growth arrest in gastric cancer cells 88. In cervical cancer, DACT1 was identified as a target gene of the lysine-specific histone demethylase 1A (KDM1A) 38. KDM1A could downregulate DACT1 expression through histone deacetylation to enhance the proliferation of cervical cancer cells 38. Moreover, Shi et al. demonstrated that H1FX-AS1 served as a ceRNA of miR-324-3p to upregulate the DACT1 expression, which induced inhibition of the proliferation of cervical cancer 37. Similarly, DACT1 was also negatively regulated by miR-324-3p, and DACT1 inhibited HCC growth by decreasing the accumulation of both cytoplasmic and nuclear β-catenin and expression of c-Myc and cyclin D1 66.

DACT2 has also been reported to be frequently down-expressed in multiple human cancers, which suppressed the proliferation of cancer cells. Restoration of DACT2 expression cloud suppresses human breast cancer cells growth through inducing G1/S checkpoint arrest 82. The expression of cyclinD1 and cyclinE1, which were critical proteins for G1‐S progression, were decreased through inhibiting the Wnt/β-catenin signaling pathway after DACT2 overexpression 35, 82, 83. Similarly, DACT2 knockdown in HCC cells and papillary thyroid cancer induced G1/S arrest of cell cycle and significant suppression of cell growth, as well as in lung cancer 45, 46. Our previous study proved that proliferation was inhibited, and G1/S arrest was enhanced by overexpression of DACT2 in glioma cells 16. Other mechanism research found the expression of PCNA and cyclinD1 were decreased after overexpression of DACT2, and restoration of DACT2 can suppress upregulation of p-YAP and prevent YAP translocating into the nucleus and sequestering in the cytoplasm to degrade through inactivation of Wnt/β-catenin signaling pathway 16. According to previous researches, DACT2 was regulated by various miRNAs to participate in the regulation of the proliferation of tumors. MiR-503-3p derived from macrophage directly targeted on DACT2 69. Reduction of miR-503-3p repressed glycolysis and promoted mitochondrial oxidative phosphorylation in breast cancer cells and decreased tumor growth by overexpressing DACT2 and inactivating the Wnt/β-catenin signaling pathway 69. Hypoxia-induced exosomal miR-181a from papillary thyroid cancer targeted and inhibited MLL3, leading to the downregulation of DACT2, which contributed to tumor growth 70. However, it was demonstrated that re-expression of DACT2 inhibited the expression of cyclinB1 and the cyclin B1-Cdk1 (Cyclin-Dependent Kinase 1, also known as cell division control protein kinase 2, CDC2) complex CDC2, and increased the levels of p-CDC2 (Y15) in esophageal and gastric cancers by inhibiting Wnt signaling pathway 51, 53. Additionally, Zhang et al. also reported that DACT2-restored expression reduced p-Smad2/3, an index of TGF-β activity, via both proteasome and lysosomal pathways, which cloud induce G2/M phase arrest in esophageal cancer 90.

As a member of the DACT family, DACT3 is also involved in tumor growth. DACT3 transfection inhibited c-Myb expression of NSCLC cells, as well as c-Myc expression and β-catenin nuclear translocation to inhibit cell proliferation 56. Over-expression of miR-31 significantly enhanced proliferation by direct interaction with Dickkopf-1 and DACT-3 in lung cancer 72.

Interestingly, DACTs exerted different roles in the regulation of colorectal cancer cell progression. It was found that DACT1 increased the nuclear and cytoplasmic fractions of β-catenin via phosphorylated GSK-3β at Ser9 to promote cell proliferation in colon cancer 40. However, reactivating DACT2 transcription significantly inhibited nuclear β-catenin expression to inactivate the Wnt/β-catenin pathway, which consequently restricted colorectal cancer cells proliferation 15, 84.

## DACTs family members regulate cancer cell apoptosis

Resistance to apoptosis is a remarkable hallmark of malignancies. Apoptosis is a programmed cell death, which is critical for removing unessential cells like tumor cells. Specific mechanisms of apoptosis in anti-carcinogenic action include activating poly (ADP-ribose) polymerase (PARP), subsequently fragments of effector caspases, and the Bcl-2 (B-cell lymphoma 2) family of proteins 92, 93. Zhu et al. demonstrated that DACT1-transfected KG-1α leukemia cells had increased expression of pro-apoptotic proteins Bax and decreased expression of anti-apoptotic proteins Bcl-2, which revealed that DACT1 might activate intrinsic apoptotic pathways 89. It is reported that re‐expression of DACT1 in breast and gastric cells appears to induce apoptosis through a caspase-dependent pathway, including activation of caspase-9, followed by cleavage of downstream caspase effectors caspase-3 and caspase-7, ultimately stimulating the activation of PARP and cellular disassembly and apoptosis 19, 25.

Moreover, DACT1 downregulated anti-apoptotic genes Bcl2 and Bcl-XL, which contributed to the prevention of mitochondrial apoptosis, leading to activation of the downstream apoptotic protease cascade 19. Similar to DACT1, ectopic expression of DACT2 led to a significant increase of apoptotic cells 15, 16, 91, induction on the expression of Bax in glioma cells 16, and the enhanced cleavage of PARP in colon cancer cells 15. In addition, it is suggested that overexpression of DACT3 resulted in a dramatic activation of caspase 3 in colorectal cancer cells and induced a sharp drop in the mitochondrial transmembrane potential that is characteristic of apoptosis 15.

Regarding the signaling pathways implicated in the regulation of cell apoptosis, DACTs have been proved to promote cell apoptosis through blocking Wnt/β-catenin 14, 15, 25, 89, 91, NF-κB 19, YAP 16 signaling pathways.

## DACT family members regulate cancer cell migration and invasion

Cell migration and invasion are central to morphogenesis and to multiple aspects of tumor metastasis 94, 95. Matrix metalloproteinases (MMPs) are a family of zinc‐containing endopeptidases collectively capable of degrading extracellular matrix components. Among the members of the MMP family, MMP-2 and MMP-9 have been widely studied and linked to increased invasion ability in various cancers 96, 97. It was reported that DACTs could inhibit the invasion and metastasis through decreasing expression and activity of MMP-2 and MMP-9 in gastric cancer 53, 98, colorectal cancer 84, esophageal cancer 51. EMT is characterized by the loss of cell-cell adhesions and gain of migratory and invasive traits, which governs tumor cell metastasis 99. The molecular characteristics of EMT contain the suppression of epithelial markers (e.g., E-cadherin) and the concomitant promotion of mesenchymal markers such as N-cadherin, Fibronectin DACT2 directly interacts with β-catenin, and Vimentin 100. Wang et al. revealed that DACT2 upregulated the expression of E-cadherin, which was enriched in the cellular membrane, especially at cell-cell contact region, indicating that DACT2 restored E-cadherin junction stability. Moreover, on E-cadherin knockdown, cell invasive ability was significantly increased in DACT2-expressing colon cancer cells, indicating that E-cadherin blockade could partially relieve the anti-metastatic potential of DACT2 15. In addition, forced transfection of DACT2 effectively reversed EMT to mesenchymal-to-epithelial transition in breast cancer cells, resulting in the upregulation of E-cadherin and downregulation of Vimentin 83.

**Figure 1:**
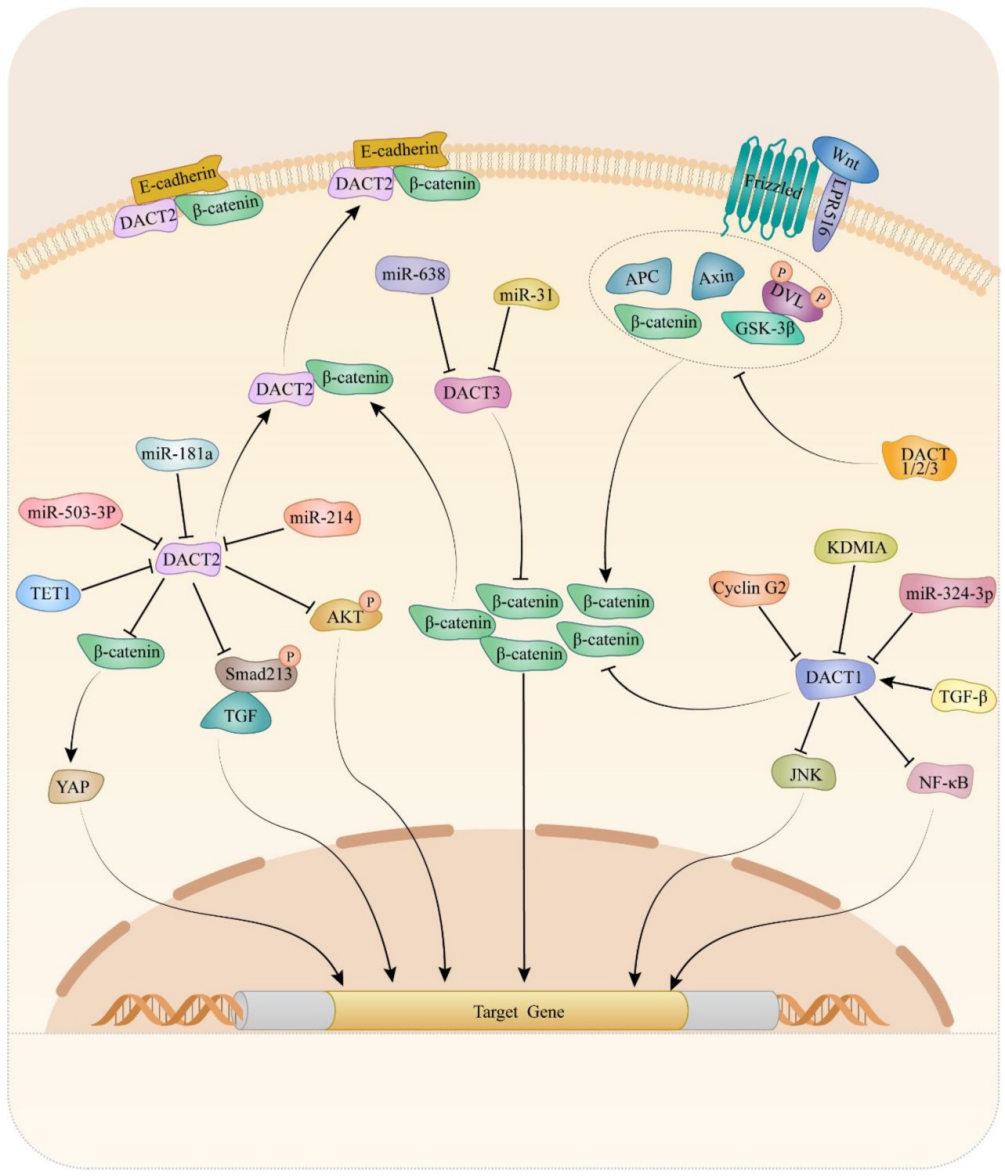
Molecular mechanism of DACTs in tumors.

Mechanically, DACTs exert its migration- and invasion-inhibitory effects through Wnt/β-catenin 15, 25, 35, 51, 53, 54, 73, 83, 88, 91, planar cell polarity (PCP) 98, TGF-β/Smad2/3 49 and Akt/GSK-3 signaling pathway 83. It is suggested that cyclin G2 could interact with DACT1 and inhibit the ability of CKI to phosphorylate DACT1, thereby stimulating β-catenin degradation in a GSK-3β-dependent in gastric cancer 88. Increasing evidence has revealed critical contributions of the PCP pathway to tumor metastasis 101-103. Liu et al. reported that DACT1 regulated the PCP pathway by promoting Dvl-2 degradation and suppressing the active form of JNK in gastric cancer cells 98. Another study indicated that restored expression of DACT2, the activity of TGF-β/Smad2/3 was suppressed via both proteasome and lysosomal degradation pathways, leading to F-actin rearrangement that might depend on the involvement of cofilin and ezrin-redixin-moesin protein in ESCC 49. Moreover, the data from breast cancer research indicated that expression of p-AKT and p-GSK-3β dramatically decreased in breast cancer cells upon DACT2 re-expression, which indicated DACT2 might regulate Akt/GSK-3 signaling pathway to involve in migration and invasion 83. Up to now, the Wnt/β-catenin signaling pathway is the most well-studied pathway regulated by DACTs. Enormous studies provided evidence that DACTs antagonize Wnt/β-catenin signaling by decreasing active β-catenin levels in cancers. In colon cancer, DACT2 binding to nuclear β-catenin, preventing it from forming a complex with its partner lymphoid enhancer-binding factor 1, was an important mechanism for DACT2-mediated Wnt/β-catenin signaling inhibition 15. Consistently, DACT3 interacted with and down-regulated Dvl2 protein and attenuated the Wnt-responsive Top flash reporter expression, which agrees with the inhibitory effect of DACT3 on Wnt signaling in colorectal cancer. As shown in Table 1 and Figure 1, different mechanisms of DACTs in regulating progression of cancers are summarized.

## DACT family members act as the positive regulator to enhance autophagy

Autophagy is a regulated self-eating process that eliminates the cellular materials, such as aggregated cytoplasmic proteins or aged and damaged organelles through lysosomal degradation 104. It has been shown autophagy is tightly involved in the Wnt/β-catenin pathway, and DACT1 and DACT3 are positive regulators to enhance autophagy. Ruo-nan Li et al. found DACT1 enhanced autophagy which enhances LC3 lipidation and p62/SQSTM1 degradation 105. Mechanistic studies suggest that DACT1 enhanced the Atg14L-Beclin1-Vps34 complex formation to drive autophagy 106. DACT1 acts as an adaptor to increase the ubiquitination of Dvl2 mediated by the von Hippel-Lindau tumor suppressor and mediates the Vps34-Beclin1 complex formation induced by protein aggregates under starvation in turn 107. In addition, literature reports that miR-638 regulates autophagy of ESCC and BC cells. Yanli Ren et al. elucidated that miRNA-638 promotes autophagy and malignant phenotypes of cancer cells via directly suppressing DACT3 104. Unfortunately, there is no more research on the mechanism of DACT3 in autophagy. It needs to further investigate the molecular mechanism of DACT3 in cancer cell autophagy and its potential therapeutic implications.

## Conclusion and future vision

In this review, we have sought here to synthesize the advances in the DACTs and their roles in various cancers and highlighted the function and mechanisms of DACTs in cancer cell proliferation, apoptosis, migration, and invasion, and autophagy, which may contribute to forming an elementary framework for understanding the complex biologic changes induced by the DACT family, and help us to develop more targeted implementation strategies. Until now, research on inhibiting or promoting cancer progression by regulating DACTs has remained in the experimental stage. We hope continuing elucidation of cancer pathogenesis, including precise molecular mechanism, to make most utility of the DACT family members.

In clinical practice, DACTs expression is associated with clinicopathological parameters and survival of tumor patients. On this basis, DACT could act as a diagnostic and prognostic biomarker for cancer patients. Nevertheless, confirming the prognostic and diagnostic value of DACTs in cohort studies with well-designed and larger-size needs to be conducted. Further exploring the role of DACTs in more cancer and whether DACTs can serve as a drug target may have important research value. We hope that our review will attract the attention of the scientific research community and clinical practitioners and encourage them to carry out treatments targeting DACTs as soon as possible.

## Figures and Tables

**Table 1:** The expression and molecular mechanism of DACTs in tumors

Molecules	Experiment subjects	Levels	Pathway	Properties	References
DACT1	Gastric Cancer	Downregulate	N/A	Be associated with the poorer survival	81
DACT1	Gastric Cancer	Downregulate	Nuclear Factor-*κ*B signaling	Inhibits gastric cancer cell growth	19
DACT1	Gastric Cancer	Downregulate	N/A	Plays a pivotal role as a potential tumor suppressor in migration and invasion of gastric cancer.	98
DACT1	Gastric Cancer	N/A	Wnt/β-catenin signaling	Cyclin G2 suppresses Wnt/β-catenin signaling and inhibits gastric cancer cell growth and migration through DACT1	88
DACT1	Bladder urothelial carcinoma	Downregulate	N/A	Implicates in carcinogenesis and the progression	36
DACT1	Cervical cancer	Downregulate	N/A	H1FX-AS1 inhibites cervical cancer cell proliferation, migration and invasion, while induces apoptosis by sponging miR-324-3p to up-regulate the DACT1 expression level	37
DACT1	Cervical cancer	Downregulate	N/A	KDM1A can downregulate DACT1 expression through histone deacetylation and therefore suppress the proliferation and migration of cervical cancer cells	38
DACT1	Breast cancer	Downregulate	Wnt/β-catenin signaling	Inhibits proliferation and migration	25
DACT1	Non-small cell lung cancer tissues	Downregulate	N/A	Relates to the clinicopathological factors and is an independent risk factor for prognosis of the patients with NSCLC	42
DACT1	Esophageal squamous carcinoma	Downregulate	N/A	Inhibits proliferation and migration	50
DACT1	Hepatocellular Carcinoma	Downregulate	Wnt/β-catenin signaling	Related to the tumorigenesis and leads to cytoplasmic accumulation of β-catenin	80
DACT1	Hepatocellular Carcinoma	N/A	Wnt/β-catenin signaling	miR-324-3p directly targets DACT1 and negatively regulates its expression in HCC cells	66
DACT1	Type I ovarian cancer	Downregulate	Wnt/β-catenin signaling	Inhibits ovarian cancer growth, tumorigenesis, and induce autophagy	39
DACT1	Leukemia	N/A	Wnt/β-catenin signaling	Inhibits Wnt/β-catenin signaling by reducing nuclear β-catenin levels and reduces P-glycoprotein expression in KG-1α cells	89
DACT1	Bone metastasis of breast and prostate	N/A	Wnt/β-catenin signaling	TGF-β induces DACT1to form protein condensates in the cytoplasm to repress Wnt signaling	73
DACT2	Colon cancer	Downregulate	Wnt/β-catenin signaling	Suppresses cell apoptosis and inhibiting cell proliferation both *in vitro* and *in vivo*	15
DACT2	Glioma	Downregulate	Wnt/β-catenin and YAP signaling	Inhibits proliferation, cell cycle and enhance apoptosis, sensitivity to temozolomide, and suppress tumor growth	16
DACT2	Breast cancer	Downregulate	Wnt/β-catenin signaling	Suppresses proliferation, invasion, tumor growth, migration, and invasion	35
DACT2	Breast cancer	Downregulate	Wnt/β-catenin signaling	Inhibits the breast cancer cells proliferation and invasion	35
DACT2	Breast cancer	NA	Wnt/β-catenin signaling	MiR-503-3p represses glycolysis and promotes mitochondrial oxidative phosphorylation in breast cancer by elevating DACT2	69
DACT2	Breast cancer	Downregulate	Wnt/β-catenin signaling	Suppresses breast cancer cell growth, induces G1/S phase arrest, inhibits Wnt/βcatenin signaling and suppresses breast cancer cell tumor growth in xenograft mice	82
DACT2	Breast cancer	Downregulate	Wnt/β-catenin signaling	Induces breast cancer cell apoptosis *in vitro*, and further inhibites breast tumor cell proliferation, migration and EMT, through antagonizing Wnt/β-catenin and Akt/GSK-3 signaling.	83
DACT2	Gastric cancer	Downregulate	Wnt/β-catenin signaling	MiR‑214 promotes the proliferation, migration and invasion of mixed gastric adenocarcinoma type gastric cancerMKN28 cells by suppressing the expression of DACT2	52
DACT2	Gastric cancer	Downregulate	Wnt/β-catenin signaling	Inhibits cell proliferation, migration and invasion in gastric cancer cells and suppresses gastric cancer xenografts in mice	53
DACT2	Prostate Cancer	Downregulate	N/A	Suppresses prostate cancer cells migration and invasion	55
DACT2	Hepatocellular Carcinoma	Downregulate	Wnt/β-catenin signaling	Suppresses cell proliferation and inhibit tumor growth	47
DACT2	Hepatocellular Carcinoma	Downregulate	Wnt/β-catenin signaling	Suppresses liver cancer progression	46
DACT2	Lung Cancer	Downregulate	Wnt/β-catenin signaling	Increases the anti-proliferation effect of gefitinib on NSCLC cells	44
DACT2	Lung Cancer	Downregulate	Wnt/β-catenin signaling	Inhibits tumor growth	45
DACT2	Esophageal squamous	Downregulate	TGF-βsignaling	Inhibits tumor growth, migration, invasion and reduce tumorigenicity	49
DACT2	Esophageal squamous	Esophageal squamous	N/A	Inhibits proliferation and migration	50
DACT2	Esophageal squamous	Esophageal squamous	Wnt/β-catenin signaling	Suppresses colony formation, cell migration, invasion in esophageal cancer cells, esophageal cancer cell xenograft growth, and Wnt signaling in human esophageal cancer cells	51
DACT2	Papillary Thyroid cancer	Downregulate	Wnt/β-catenin signaling	Suppresses cell proliferation, invasion, and migration	54
DACT2	Papillary Thyroid cancer	N/A	N/A	MiR-181a inhibits DACT2 by downregulating MLL3, leading to YAP-VEGF-mediated angiogenesis	70
DACT2	Nasopharyngeal carcinoma	Downregulate	Wnt/β-catenin signaling	Inhibits nasopharyngeal carcinoma cell proliferation and metastasis	85
DACT3	Colon cancer	Downregulate	Wnt/β-catenin signaling	Leads to inhibition of Wnt/β-catenin signaling and massive apoptosis of colorectal cancer cells	14
DACT3	Non-small cell lung cancer	Downregulate	Wnt/β-catenin signaling	Inhibits the malignant phenotype of non-small cell lung cancer	56
DACT3	Esophageal squamous	Downregulate	N/A	Unknown	50
DACT3	Esophageal squamous	N/A	N/A	MiR-638 regulates expression of DACT3 and promotes autophagy as well as malignancy	71
DACT3	Lung Cancer	N/A	N/A	MiR-31 diminishes Dkk-1 and DACT3 expression levels in normal respiratory epithelia and lung cancer cells	72

N/A: Not Applicable.

**Table 2:** miRNAs involved in the regulation of DACTs expression

Molecules	MicroRNAs	Properties	References
DACT1	miR-324-3p	MiR-324-3p negatively regulates DACT1 expression in HCC cells, and lncRNA H1FX-AS1 can act as a competing endogenous RNA of miR-324-3p to inhibit DACT1 cervical cancer progression	37, 66
DACT2	miR-214	MiR-214 inhabits DACT2 expression in gastric cancer tissues to promote the occurrence and development of gastric cancer	52
	miR-503-3p	DACT2 increases accompanied by decreased expression of miR-503-3p in breast cancer tissues	69
	miR-181a	MiR-181a inhibits DACT2 by downregulating MLL3 expression, resulting papillary thyroid cancer progression	70
DACT3	miR-638	MiR-638 negatively regulates expression of DACT3 to promote cancer malignancy	71
	miR-31	MiR-31 directly interacts with DACT-3 to decrease the expression of DACT3 in lung cancer cells	72

**Table 3:** DACT family commonly methylated in cancers

Tissue	Gene	Assay	Methylation prevalence (%)			References
			Normal	Para-cancer	cancer	
Breast cancer	DACT1	MSP	0% (0/15)	0% (0/11)	29.9% (40/134)	25
Breast cancer	DACT2	MSP	N/A	0% (0/15)	32.9% (26/79)	43
Breast cancer	DACT2	MSP	N/A	N/A	83% (10/12)	35
Breast cancer	DACT2	MSP	N/A	66.7% (16/24)	49.7% (76/153)	82
Breast cancer	DACT2	MSP/BGS	0% (0/14)	20% (1/5)	73% (107/147)	83
Bladder urothelial carcinoma	DACT1	MSP	25% (7/29)	N/A	58.62% (17/29)	36
Prostate cancer	DACT2	MSP/BGS	N/A	N/A	68.1% (32/47)	55
Gastric cancer	DACT1	MSP	N/A	N/A	29.3% (60/205)	19
Gastric cancer	DACT1	MSP	0% (0/25)	N/A	28.3% (25/459)	81
Gastric cancer	DACT1	MSP	0% (0/20)	N/A	29.3% (60/205)	98
Gastric cancer	DACT2	MSP	0% (0/8)	N/A	55.7% (93/167)	53
Nasopharyngeal cancer	DACT1	MSP	N/A	N/A	70.9% (44/62)	86
Nasopharyngeal cancer	DACT2	MSP	0% (0/8)	N/A	91% (29/32)	85
Colon cancer	DACT2	BGS	0% (0/12)	N/A	43.3% (29/67)	15
	DACT2	MSP	N/A	N/A	46% (23/50)	48
Esophageal squamous	DACT1	MSP/BGS	N/A	16.4%-47.8% (26/159-76/159)	43.4%-54.1% (69/159-86/159)	50
Esophageal squamous	DACT2	MSP/BGS	N/A	21.4% (34/159)	52.3% (83/159)	50
Esophageal squamous	DACT2	MSP/BGS	0% (0/27)	N/A	69% (87/126)	51
Esophageal squamous	DACT3	MSP/BGS	N/A	3.8%	5.7%	50
Hepatocellular carcinoma	DACT1	MSP	0% (0/3)	18% (9/43)	51% (22/43)	80
Hepatocellular carcinoma	DACT2	MSP	N/A	N/A	54.84% (34/62)	47
Lung cancer	DACT2	MSP	0% (0/4)	N/A	41% (43/106)	45
Papillary thyroid cancer	DACT2	MSP	0% (0/10)	N/A	64.6%	54
Oral squamous carcinoma	DACT2	MSP	N/A	N/A	70.2% (33/47)	86
Bladder urothelial carcinoma	DACT1	MSP	N/A	25% (7/29)	58.62% (17/29)	36
Breast cancer	DACT1	MSP	0% (0/15)	0% (0/11)	29.9%(40/134)	25
	DACT2	MSP	N/A	0% (0/15)	32.9% (26/79)	43
		MSP	N/A	N/A	83% (10/12)	35
		MSP	N/A	66.7% (16/24)	49.7%(76/153)	82
		MSP/BGS	0% (0/14)	20% (1/5)	73% (107/147)	83
Bladder urothelial carcinoma	DACT1	MSP	25% (7/29)	N/A	58.62% (17/29)	36
Prostate cancer	DACT2	MSP/BGS	N/A	N/A	68.1% (32/47)	55
Gastric cancer	DACT1	MSP	N/A	N/A	29.3% (60/205)	19
		MSP	0% (0/25)	N/A	28.3% (25/459)	81
		MSP	0% (0/20)	N/A	29.3% (60/205)	98
	DACT2	MSP	0% (0/8)	N/A	55.7% (93/167)	53
Nasopharyngeal cancer	DACT1	MSP	N/A	N/A	70.9% (44/62)	86
	DACT2	MSP	0% (0/8)	N/A	91% (29/32)	85
Colon cancer	DACT2	BGS	0% (0/12)	N/A	43.3% (29/67)	15
	DACT2	MSP	N/A	N/A	46% (23/50)	48
Esophageal squamous	DACT1	MSP/BGS	N/A	16.4%-47.8% (26/159-76/159)	43.4%-54.1% (69/159-86/159)	50
	DACT2	MSP/BGS	N/A	21.4% (34/159)	52.3% (83/159)	50
		MSP/BGS	0% (0/27)	N/A	69% (87/126)	51
	DACT3	MSP/BGS	N/A	3.8%	5.7%	50
Hepatocellular carcinoma	DACT1	MSP	0% (0/3)	18% (9/43)	51% (22/43)	80
	DACT2	MSP	N/A	N/A	54.84% (34/62)	47
Lung cancer	DACT2	MSP	0% (0/4)	N/A	41% (43/106)	45
Papillary thyroid cancer	DACT2	MSP	0% (0/10)	N/A	64.6%	54
Oral squamous carcinoma	DACT2	MSP	N/A	N/A	70.2% (33/47)	86
Bladder urothelial carcinoma	DACT1	MSP	N/A	25% (7/29)	58.62% (17/29)	36

## Abbreviations

DACT: Dishevelled-associated antagonist of β-catenin
TSGs: tumor suppressor genes
Dvl: Dishevelled
NES: nuclear export signal
NLS: nuclear localization signal
EMT: epithelial-mesenchymal transition
NSCLC: non-small cell lung cancer
ESCC: esophageal squamous cell carcinoma
HCC: hepatocellular carcinoma
miRNAs: MicroRNAs
KDM1A: histone demethylase 1A
MLL3: mixed-lineage leukemia 3
CGIs: CpG islands
MSP: Methylation-specific PCR
BGS: bisulfite genomic sequencing
PARP: poly (ADP-ribose) polymerase
Bcl-2: B-cell lymphoma 2
MMP: Matrix metalloproteinases
PCP: planar cell polarity
